# Altered resting-state functional connectivity within corticostriatal and subcortical-striatal circuits in chronic pain

**DOI:** 10.1038/s41598-022-16835-7

**Published:** 2022-07-25

**Authors:** Su Hyoun Park, Anne K. Baker, Vinit Krishna, Sean C. Mackey, Katherine T. Martucci

**Affiliations:** 1 Department of Anesthesiology, Duke University Medical Center, Durham, USA; 2 Duke Center for Translational Pain Medicine, Durham, USA; 3 Department of Anesthesiology, Perioperative, and Pain Medicine, Stanford University School of Medicine, Stanford, USA; 4 Human Affect and Pain Neuroscience Lab, Department of Anesthesiology, Duke University Medical Center, Box DUMC 3094, Durham, NC 27710 USA

**Keywords:** Medical research, Neurology, Rheumatology, Cognitive neuroscience, Diseases of the nervous system, Neural circuits, Reward

## Abstract

Brain corticostriatal circuits are important for understanding chronic pain and highly relevant to motivation and cognitive processes. It has been demonstrated that in patients with chronic back pain, altered nucleus accumbens (NAcc)—medial prefrontal cortex (MPFC) circuit fMRI-based activity is predictive of patient outcome. We evaluated the NAcc-MPFC circuit in patients with another chronic pain condition, fibromyalgia, to extend these important findings. First, we compared fMRI-based NAcc-MPFC resting-state functional connectivity in patients with fibromyalgia (N = 32) vs. healthy controls (N = 37). Compared to controls, the NAcc-MPFC circuit’s connectivity was significantly reduced in fibromyalgia. In addition, within the fibromyalgia group, NAcc-MPFC connectivity was significantly correlated with trait anxiety. Our expanded connectivity analysis of the NAcc to subcortical brain regions showed reduced connectivity of the right NAcc with mesolimbic circuit regions (putamen, thalamus, and ventral pallidum) in fibromyalgia. Lastly, in an exploratory analysis comparing our fibromyalgia and healthy control cohorts to a separate publicly available dataset from patients with chronic back pain, we identified reduced NAcc-MPFC connectivity across both the patient groups with unique alterations in NAcc-mesolimbic connectivity. Together, expanding upon prior observed alterations in brain corticostriatal circuits, our results provide novel evidence of altered corticostriatal and mesolimbic circuits in chronic pain.

## Introduction

Motivation and cognitive processes are central to human survival^1,2^ and powerfully modulate the experience of pain^3,4^. Such processes involve brain regions including the nucleus accumbens (NAcc), amygdala, hippocampus, thalamus, anterior cingulate cortex (ACC), and medial prefrontal cortex (MPFC)^5^. Connections between these regions form a complex cortico-basal ganglia network to support human behaviors and cognition^5^. Chronic pain is often comorbid with dysfunctional cognitive and motivation processes^3,6,7^, and the cortico-basal ganglia network plays an important role in chronic pain^8,9^.

Prior functional magnetic resonance imaging (fMRI) studies have used a measure of functional connectivity (i.e., correlation of blood oxygenation level dependent [BOLD] signal activity between neuroimaged regions)^10^ to investigate circuits within the cortico-basal ganglia network^11^, with a specific focus on corticostriatal circuits^12,13^. Studies using such methods have shown that patients with chronic pain demonstrate evidence of dysfunctional corticostriatal (i.e., NAcc-MPFC) circuits^12,13^. For example, when patients with chronic back pain receive thermal stimulation and perform a coinciding self-report pain-monitoring task, compared to healthy controls, patients exhibit greater functional connectivity between the NAcc and MPFC regions^12^. As revealed in another study when patients with sub-acute back pain performed a continuous self-report pain-monitoring task during an otherwise resting-state fMRI scan, functional connectivity between the NAcc and MPFC predicts pain chronification. (i.e., greater NAcc-MPFC functional connectivity is observed in patients whose pain persists vs. patients who recover)^13^. However, no studies have evaluated this specific NAcc-MPFC circuit in patients with back pain (or other types of chronic pain) during resting-state fMRI with no task involved.

In addition to such findings in patients with back pain, corticostriatal activity is similarly altered in other chronic pain conditions, including fibromyalgia. Fibromyalgia is a condition of widespread chronic pain, that is typically accompanied by cognitive-, mood-, and fatigue-related symptoms^14^. Compared to healthy controls, patients with fibromyalgia demonstrate greater MPFC activity during avoidance of punishment, and reduced MPFC activity during anticipation of reward^15,16^. Similarly, patients with fibromyalgia demonstrate reduced midbrain activity both during anticipation of pain (i.e., punishment) and pain relief (i.e., rewarding event)^17^. Likewise, in patients with chronic pain (mixed cohort of fibromyalgia or chronic low back pain), the right striatum shows reduced response during anticipation of reward and loss^18^. Thus, based on these multiple lines of evidence of altered activity within corticostriatal brain regions, it follows that corticostriatal circuits, specifically of the NAcc-MPFC, would be altered in patients with fibromyalgia, as similar to patients with chronic back pain.

To extend prior evidence of altered corticostriatal circuits to new cohorts of chronic pain patients, in the present study, we aimed to evaluate the NAcc-MPFC circuit in patients with fibromyalgia and to further evaluate activity within subcortico-striatal networks. Importantly, we evaluated these circuits by analyzing resting-state fMRI with no task involved. As outlined in our pre-registered plan on the Open Science Framework (OSF, https://osf.io/cj9u8), we hypothesized that resting-state corticostriatal circuits (i.e., measured as fMRI-indicated NAcc-MPFC connectivity) are altered in patients with fibromyalgia. Therefore, to test our hypothesis, we first examined corticostriatal connectivity strength of the NAcc-MPFC circuit in patients with fibromyalgia compared to healthy controls. Additionally, in our fibromyalgia cohort, we probed relationships between NAcc-MPFC connectivity and clinical measures of mood, affect, and symptom severity. Then, we conducted an expanded analysis of NAcc resting-state functional connectivity to mesolimbic (i.e., subcortical) circuit regions in fibromyalgia. In addition, as an exploratory analysis, we compared our findings (i.e., corticostriatal and subcortical-striatal connectivity of patients with fibromyalgia and healthy controls) with another chronic pain condition, chronic back pain (CBP), using publicly available dataset (openpain.org).

## Results

### Participant demographics, medications, and clinical measures

We collected resting-state fMRI data from a total of 32 patients with fibromyalgia and 37 healthy controls. As presented in this study, the full dataset represents data collected as part of two separate studies at Stanford University (N = 17 fibromyalgia; N = 17 healthy control) and Duke University (N = 15 fibromyalgia; N = 20 healthy control) by the same investigator at each site (K.T.M.). (Although we had initially analyzed resting-state fMRI data from the Stanford dataset only [medRxiv preprint^19^], the combined Stanford and Duke datasets are analyzed here to increase the sample size.) We provide the demographic data in Supplementary Table S1. Among the patients with fibromyalgia, the duration of pain related to fibromyalgia symptoms ranged from 9 months to 28 years (M = 9.08 years, SD = 7.35 years). Questionnaire measures, including mood disturbance, fatigue, anxiety, depression, pain distribution across the body, pain severity, and pain interference, were significantly higher in patients with fibromyalgia compared to healthy controls in both datasets (see Supplementary Table S2). As the participants in the Stanford dataset were involved in a larger study, we have previously reported responses to a reward task from these same patients as published elsewhere^15,16^. However, we have not previously described the resting-state fMRI data from the Stanford and Duke data sets other than in the preprint. In an exploratory analysis, we included resting-state fMRI data from a cohort of 31 chronic back pain patients (publicly available data, openpain.org). From the chronic back pain data set (openpain.org), demographic and clinical data were only partially available at the time of analysis (Supplementary Table S1). For patients with chronic back pain, the duration of pain ranged from 1 to 38 years (M = 15.35 years, SD = 10.78 years).

### NAcc-MPFC circuit functional connectivity in patients with fibromyalgia vs. healthy controls

**Figure 1:**
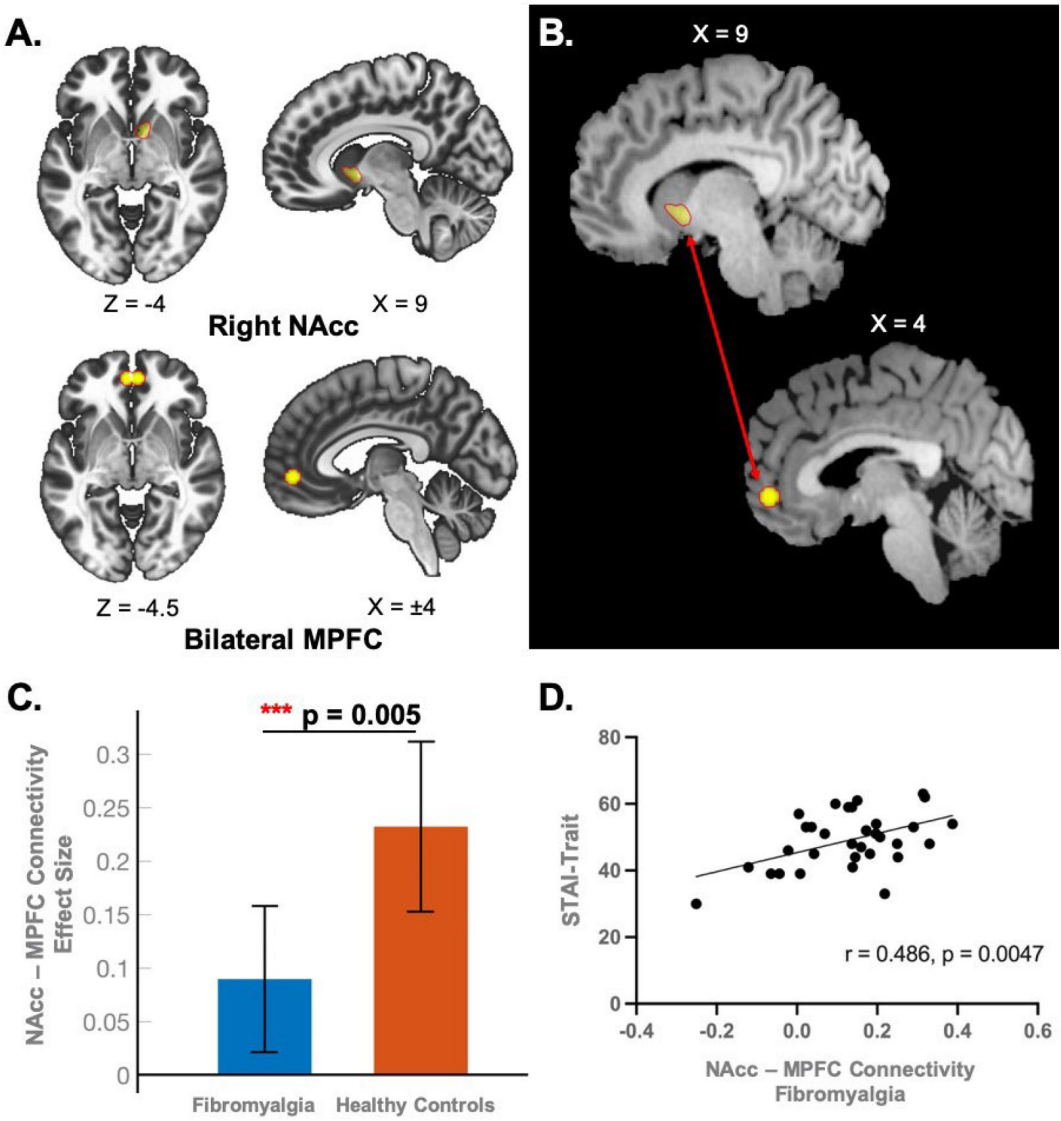
NAcc-MPFC functional connectivity in patients with fibromyalgia vs. healthy controls. (**A**) Location of the right NAcc ROI (top) and bilateral MPFC VOI (bottom). (**B**) Right NAcc ROI (top)—Bilateral MPFC VOI (bottom). (**C**) NAcc-MPFC connectivity in patients with fibromyalgia vs. healthy controls. Effect size refers the average difference in Fisher-transformed correlation coefficients. Error bars indicate the 95% confidence interval. (**D**) Correlations between NAcc-MPFC connectivity and STAI-Trait within the fibromyalgia group. ROI, region of interest, i.e., brain region defined; VOI, volume of interest, i.e., sphere at specific coordinates; NAcc, nucleus accumbens; MPFC, medial prefrontal cortex.

Our a-priori hypothesis was that NAcc-MPFC resting-state functional connectivity would be altered in patients with fibromyalgia (as demonstrated in prior investigations of patients with chronic back pain). Therefore, we first aimed to identify group differences in NAcc-MPFC connectivity in patients with fibromyalgia compared to healthy controls (Fig. 1A,B). Since the duration of pain can impact the heterogeneity of functional connectivity^20^ and these datasets were collected across two study sites, we included pain duration and study site as covariates. In addition, due to a significant difference in patients’ age between the two sites (Stanford: M = 48.11, SD = 7.47; Duke: M = 34.86, SD = 11.65; t(26) = 3.34, p = 0.002), we also included age as covariate. For healthy controls, no significant age difference across the two sites was observed (p = 0.38). When combining the two datasets, no significant difference in age was observed between patients and controls (p = 0.09).

In both patients with fibromyalgia and healthy controls, robust NAcc-MPFC connectivity was demonstrated (i.e., significantly correlated activity between NAcc and MPFC; t(31) = 5.1, p < 0.0001 and t(36) = 8.28, p < 0.0001, respectively). Further, when comparing across fibromyalgia and control groups, NAcc-MPFC connectivity was significantly lower in fibromyalgia (t(64) = − 2.91, p = 0.005; Fig. 1C).

The right NAcc region of interest (ROI, i.e., brain region defined) and bilateral MPFC volume of interest (VOI, i.e., sphere defined at specific coordinates) were predetermined based on the approach used in Martucci et al.^15^. As both Martucci et al. and our present study have strong predetermined hypotheses (i.e., primary analyses restricted to the NAcc and MPFC), we followed this previous approach in creating the right NAcc ROI and bilateral MPFC VOI (i.e., 5-mm radius spheres centered at [± 4, 50, − 4.5]). To determine whether a slightly different ROI may lead to different patterns of results, we created two more bilateral MPFC VOIs (i.e., 8-mm and 10-mm radius spheres centered at [± 4, 50, − 4.5]) and the same VOIs used by Baliki et al.^13^ (i.e., 5-mm radius spheres centered at [10, 12, − 8] for the right NAcc VOI and at [2, 52, − 2] for the right MPFC VOI) as an exploratory analysis (see Supplementary Fig. S1 for exploratory VOIs). Then, we followed the same statistical analysis steps used for our primary analysis. Again, as confirmed by the exploratory analyses, significantly reduced NAcc-MPFC connectivity in patients vs. controls was observed for: (1) 8-mm radius sphere MPFC VOI and the NAcc ROI, t(64) = − 2.62, p = 0.011, and (2) 10-mm radius sphere MPFC VOI and the NAcc ROI, t(64) = − 2.36, p = 0.021. Additionally, when using MPFC and NAcc VOIs as in Baliki et al.^13^, a trend of group difference (i.e., reduced NAcc-MPFC connectivity in patients vs. controls) was observed, t(64) = − 1.76, p = 0.083.

For exploratory analyses using the left NAcc and bilateral NAcc as alternative NAcc ROIs (as described in our pre-registered analysis plan in OSF, https://osf.io/cj9u8), we re-tested the primary hypothesis (i.e., functional connectivity between the right NAcc and bilateral MPFC) comparing connectivity with the bilateral MPFC in patient vs. healthy control groups. While no group difference was shown with the left NAcc-bilateral MPFC connectivity, as compared to controls, patients showed significantly reduced bilateral NAcc-bilateral MPFC connectivity (t(64) = − 2.41, p = 0.018).

In correlational analyses, we assessed the extent to which right NAcc-bilateral MPFC connectivity related to clinical, affective, and cognitive measures in patients with fibromyalgia. Clinical measures included fatigue (PROMIS Fatigue), three BAS subscales (behavioral drive, behavioral reward responsiveness, and behavioral fun seeking), behavioral inhibition (BIS subscale), positive affect (PANAS, PAS subscale), negative affect (PANAS, NAS subscale), trait anxiety (STAI Trait), state anxiety (STAI State), total mood disturbance (POMS), depression (BDI), pain severity (BPI), and pain interference (BPI). As similarly performed in Martucci et al.^15^, correlations identified among clinical measures revealed 5 independent clusters of measures: (1) BAS fun, BAS reward, BAS drive, and BIS (p ≤ 0.001), (2) STAI-trait, STAI-state, BDI, and POMS (p ≤ 0.036), (3) pain severity and pain interference (BPI) (p = 0.004), (4) PROMIS Fatigue and PAS subscale (p = 0.003), and (5) NAS subscale. Accordingly, the NAcc-MPFC connectivity vs. clinical measures correlational analyses were Bonferroni corrected for a total of 6 independent comparisons (i.e., NAcc-MPFC connectivity plus 5 independent clusters of measures) and determined to be significant at the level of p < 0.008 (corrected threshold). Within the fibromyalgia patient cohort only, correlational analysis of connectivity vs. questionnaire measures showed a significant positive relationship between NAcc-MPFC connectivity and anxiety, (STAI-Trait, r = 0.486, p = 0.005) (Fig. 1D). No significant correlations were identified between NAcc-MPFC connectivity and other clinical measures (Table 1).

**Table 1:** Correlations between fMRI and questionnaire measures in fibromyalgia.

	R-value	P-value
Positive affect (PANAS)	0.078	0.673
Negative affect (PANAS)	0.248	0.171
Behavioral reward (BAS)	− 0.03	0.869
Behavioral drive (BAS)	− 0.017	0.929
Behavioral fun (BAS)	0.131	0.476
Behavioral inhibition (BIS)	0.128	0.484
Total mood disturbance (POMS)	0.071	0.7
Fatigue (PROMIS)	0.147	0.422
Trait anxiety (STAI)	0.486	0.005
State anxiety (STAI)	0.025	0.891
Depression (BDI)	0.172	0.354
Pain severity (BPI)	0.151	0.409
Pain interference (BPI)	0.108	0.557

Tested relationships between NAcc-MPFC circuit connectivity and cognitive, affective, and clinical measures within patients are shown. As described above, correlational analyses were Bonferroni corrected for a total of 6 independent comparisons and determined to be significant at the level of p < 0.008 (corrected threshold).

We conducted an additional analysis to compare our NAcc-MPFC connectivity results from patients with fibromyalgia and healthy controls to a separate dataset from a cohort of patients with chronic back pain (from the OPP database, openpain.org). Of note, these patients with chronic back pain underwent similar resting-state fMRI procedures, which did not involve any task during the scan^21,22^ [*in contrast to previous findings of increased NAcc-MPFC connectivity in patients with chronic back pain, we identified reduced NAcc-MPFC connectivity in patients with fibromyalgia. To examine whether this different pattern in results may be due to methodologic differences between our study and the previous study*^12^ *(i.e., task vs. resting-state fMRI conditions), we compared our findings above to a cohort of patients with chronic back pain under conditions of resting-state fMRI that did not involve any task during the scan*]. In this analysis, age and pain duration were included as covariates. Due to unstated information for the chronic back pain dataset regarding the number of study sites involved, study site was not included as covariate. As revealed by one-way analysis of covariance (ANCOVA), a NAcc-MPFC connectivity across three groups was significantly different, [F(2, 95) = 4.49, p = 0.013]. Further, as revealed by post-hoc t-test, NAcc-MPFC connectivity in patients with chronic back pain and fibromyalgia was significantly decreased as compared to controls (chronic back pain > controls: t(95) = − 2.16, p = 0.032; fibromyalgia > controls: t(95) = − 2.95, p = 0.004) (Supplementary Fig. S2A). Notably, the failure to replicate the previous findings^12^ suggests that performing pain-related tasks (e.g., pain monitoring task or thermal stimulation) during resting-state may influence corticostriatal connectivity in chronic pain.

### NAcc-mesolimbic resting-state functional connectivity in chronic pain

**Figure 2:**
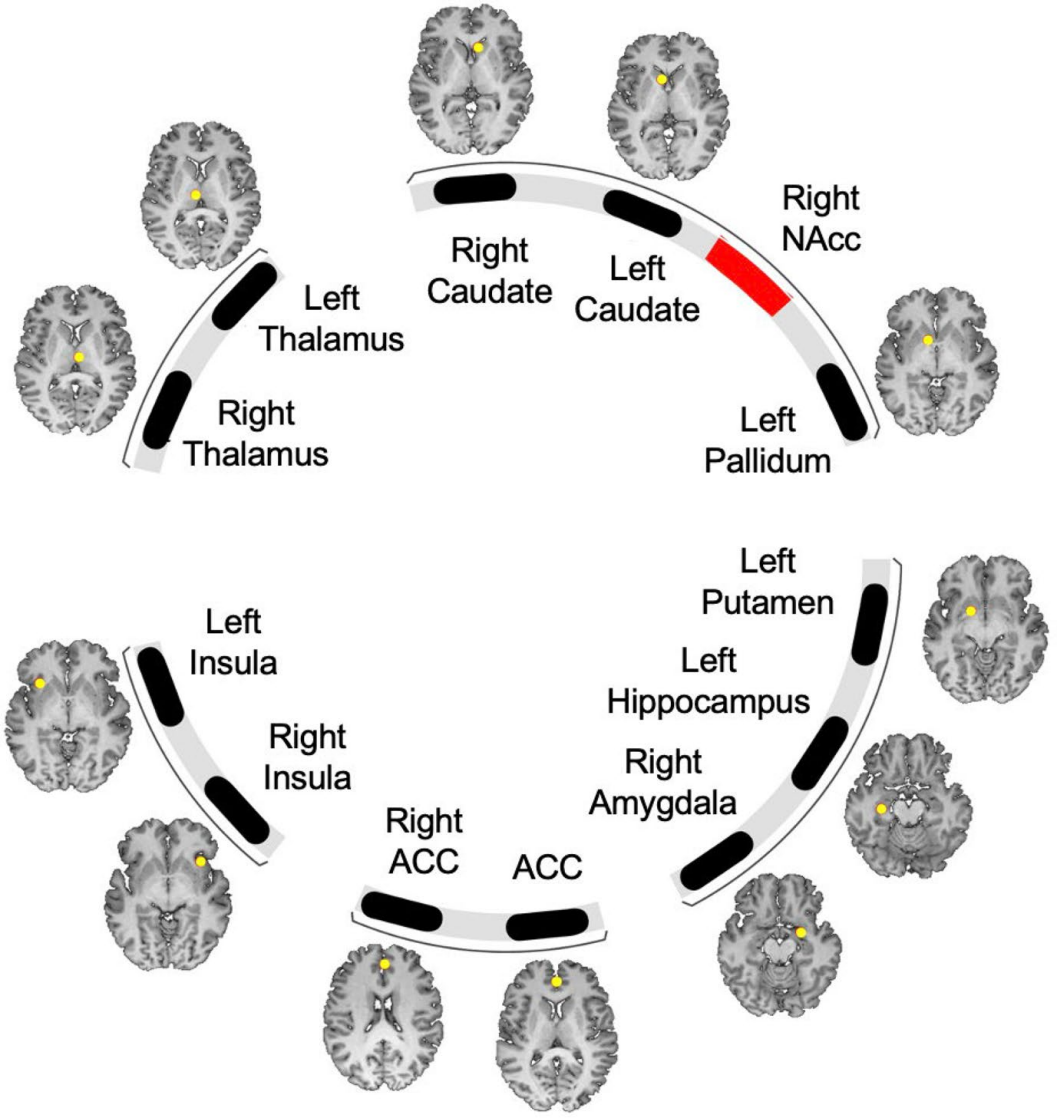
Mesolimbic volumes of interest. Location of 12 VOIs within mesolimbic circuits as defined in Liu et al.^23^ for exploratory analyses. Each of the volumes of interest were a fixed size of 10-mm diameter spheres which were centered at the left hippocampus (− 30, − 20, − 18), right amygdala (24, − 2, − 16), left and right caudate (− 8, 14, 2; 8, 20, 2), left putamen (− 16, 4, − 10), left and right insula (− 32, 20, − 4; 36, 20, − 6), left and right thalamus (− 6, − 16, 8; 4, − 14, 8), left ventral pallidum (− 10, 8, − 4), and right and central anterior cingulate cortex (2, 44, 20; 0, 44, 10). “ACC” in the figure refers to the central anterior cingulate cortex with x coordinate at 0. VOI, volume of interest; NAcc, nucleus accumbens; ACC, anterior cingulate cortex.

As described in our pre-registered analysis plan in OSF, https://osf.io/cj9u8, we measured resting-state functional connectivity between the right NAcc and several regions within the expanded mesolimbic circuit (see Fig. 2). Mesolimbic circuit regions included the ACC (x coordinate centered at 0), right ACC, left caudate, right caudate, left insula, right insula, left thalamus, right thalamus, left putamen, left hippocampus, left ventral pallidum, and right amygdala. As this was an exploratory analysis, results were not corrected for multiple comparison, however, both uncorrected and false discovery rate (FDR) corrected p-values are reported. As performed in the primary analysis, we included pain duration, age, and study site as covariates.

**Figure 3:**
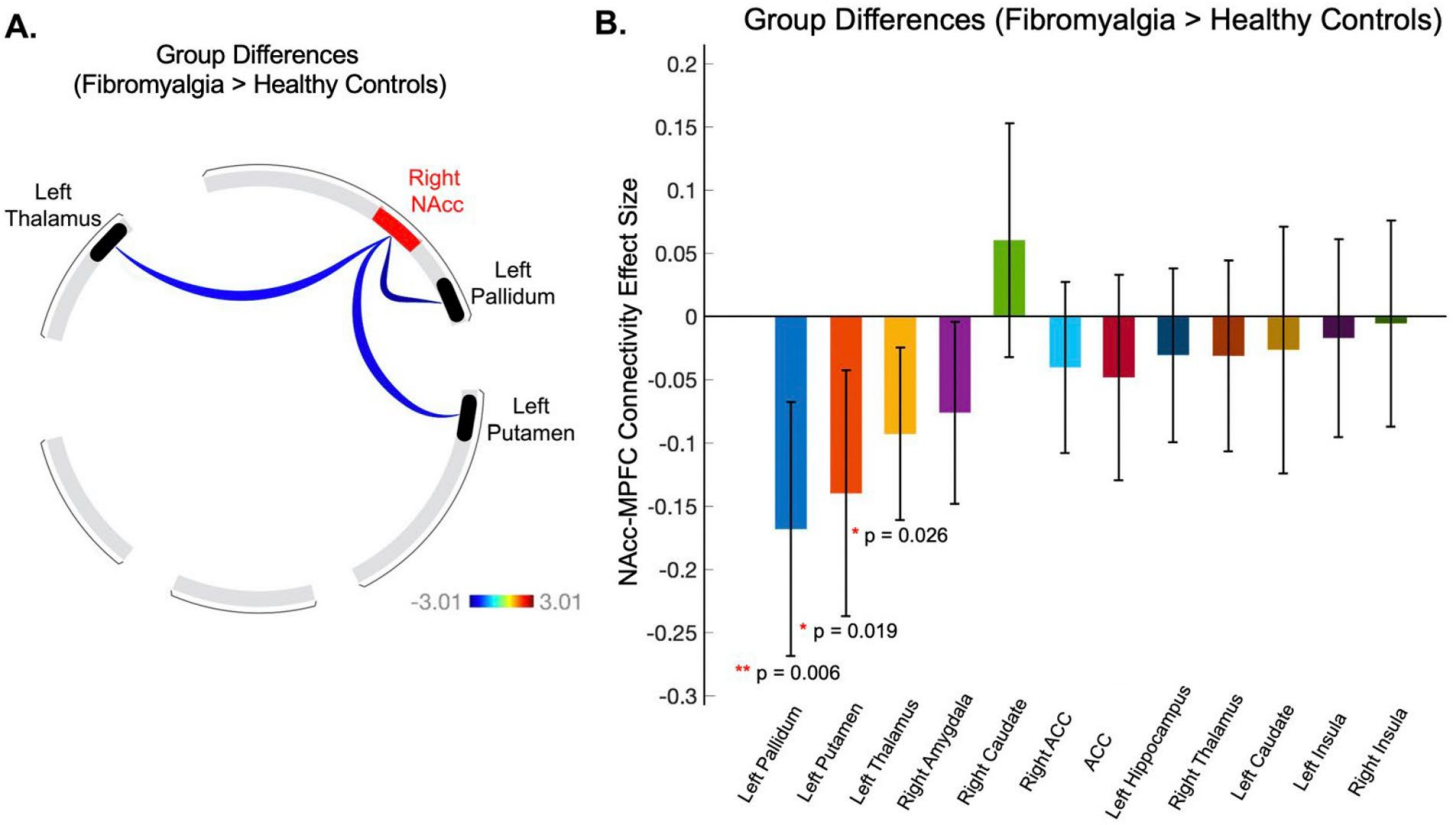
Resting-state functional connectivity between the right NAcc and 12 brain regions within mesolimbic circuits. (**A**) For the group difference, the contrast used was Fibromyalgia > Healthy Controls (e.g., reduced left putamen—right NAcc connectivity in patients with fibromyalgia compared to healthy controls). Line color indicates the magnitude of the effect for ROI-to-VOI connectivity. Blue—light blue color lines indicate negative connectivity. Results are thresholded for significance at p < 0.05 (exploratory analysis, uncorrected for multiple comparisons). (**B**) Group comparison (Fibromyalgia > Healthy Controls) of the average difference in Fisher-transformed correlation coefficients (i.e., effect sizes) for all 12 mesolimbic circuit brain regions. Error bars indicate the 95% confidence interval (uncorrected p-values). ROI, region of interest; VOI, volume of interest; NAcc, nucleus accumbens; ACC, anterior cingulate cortex.

As compared to healthy controls, patients with fibromyalgia showed decreased connectivity of the right NAcc with the left putamen (t(64) = − 2.4, p-uncorrected = 0.019, p-FDR corrected = 0.104), left thalamus (t(64) = − 2.28, p-uncorrected = 0.026, p-FDR corrected = 0.104), and left ventral pallidum (t(63) = − 2.8, p-uncorrected = 0.006, p-FDR corrected = 0.081) (Fig. 3). To further evaluate group differences between (1) chronic back pain patients (OPP) vs. fibromyalgia (our dataset) and (2) chronic back patients (OPP) vs. healthy controls (our dataset), we examined connectivity between the right NAcc and the expanded mesolimbic circuit regions across these cohorts. In chronic back pain, as compared to fibromyalgia patients and healthy controls, we observed unique patterns of subcortical-striatal connectivity alterations (see Supplementary Fig. S2B).

## Discussion

We aimed to characterize brain corticostriatal and subcortico-striatal circuits in fibromyalgia, specifically connectivity of the key NAcc-MPFC circuit and broader NAcc-mesolimbic connectivity. First, we identified robust NAcc-MPFC connectivity in both patients with fibromyalgia and healthy controls, with significant group differences. Specifically, as compared to controls, patients showed reduced NAcc-MPFC connectivity. In addition, patients with fibromyalgia demonstrated a significant correlation between NAcc-MPFC connectivity and trait anxiety. Next, through our expanded NAcc connectivity analysis including the broader mesolimbic circuits, we identified novel evidence of mesolimbic reward system alterations in chronic pain. Specifically, patients with fibromyalgia demonstrated reduced connectivity of the right NAcc with the left putamen, left thalamus, and left ventral pallidum.

Overall, our findings suggest that corticostriatal and subcortical-striatal circuit connectivity are altered during resting-state conditions in patients with fibromyalgia. As consistent with prior observations of altered brain reward response to behavioral cues in fibromyalgia^15,16^ our present results implicate corticostriatal system/circuit wide alterations in patients with fibromyalgia and chronic back pain. Extensive prior neuroimaging research has identified altered brain reward activity in patients with fibromyalgia^15–17,24^ and chronic back pain^18,25,26^. Thus, building upon prior work, our results provide further insight into how brain corticostriatal and subcortical-striatal circuits underlying such reward and motivational processes are altered in chronic pain.

Notably, in contrast to previous findings^12^, we identified reduced, rather than increased, NAcc-MPFC connectivity consistently across both of our chronic pain patient groups. In considering how prior reported increased NAcc-MPFC connectivity in patients with chronic back pain^12,13^ contrasts sharply with our present results, several factors may have contributed to the observed study differences. Sex and demographic differences may have differently influenced our results compared to previous published findings^12,13^. (For example, sex distribution in the present study was all female while sex distribution in Baliki et al.^12^ was comprised of 8 males and 8 females.) Additionally, differences in clinical status may have contributed to study differences (e.g., pain duration, our study: mean = 9.08 years vs. Baliki et al.^12^: mean = 7.86 years). More importantly, however, while administration of noxious thermal stimuli or a self-report pain-rating task were used during fMRI scans in previous functional connectivity analyses^12,13^, no task and/or stimulation experience was involved during the acquisition of resting-state fMRI data in our fibromyalgia cohort nor (to the best of our knowledge) in the publicly available chronic back pain cohort dataset. As methodologic differences may impact our comparative results, pain stimulation and/or monitoring may drive increased NAcc-MPFC functional connectivity in chronic pain conditions vs. healthy controls^12^. Consistent with this observation, in another study which demonstrated greater NAcc-MPFC functional connectivity in patients with sub-acute back pain (SBP) whose pain persisted vs. patients with SBP who recovered^13^, a self-report pain-monitoring task was involved. In line with these methodologic differences which parallel differences in results, it is reasonable to suspect that concurrent cognitive evaluation/experience with ongoing chronic pain may influence NAcc-MPFC functional connectivity. Specifically, NAcc-MPFC connectivity appears to be reduced in chronic pain states during task-free resting state, but is heightened in chronic pain conditions during self-monitoring and pain tasks^12,13^. Indeed, several research studies have identified intrinsic differences between task-based and resting-state fMRI when characterizing and differentiating brain signals and networks^27–30^. Our present results underscore that these nuanced differences (i.e., task vs. task-free resting-state conditions) are essential to consider in future evaluations of brain circuit connectivity in chronic pain.

Within the broader mesolimbic system, analysis of NAcc-subcortical circuits revealed novel significant differences in both groups of patients with fibromyalgia and patients with chronic back pain. Specifically, patients with fibromyalgia demonstrated significantly reduced connectivity between the NAcc and the left ventral pallidum, left putamen, and left thalamus. All of these brain regions play essential roles in motivational/reward processing^31–34^. Previous functional connectivity studies have revealed a critical role for thalamic projections in shaping and supporting motivational and reward processing within the NAcc among both healthy adults and adolescents^35^. Reduced connectivity between the putamen and NAcc has been demonstrated in patients with anorexia nervosa^36^, a condition highly related to brain subcortical system alterations^37–39^. Furthermore, patients with chronic back pain (vs. fibromyalgia) demonstrated reduced connectivity between the NAcc and the right caudate (Supplementary Fig. S2B). The caudate plays an essential role as a connector in shaping brain networks in patients with chronic back pain^40^. Further, the caudate is a critical region for processing pain-related information^41^ and shows reduced activation (along with the NAcc) in patients with major depressive disorder while performing a reward task^42^. Similar to these prior studies in other healthy and clinical populations, our findings of reduced connectivity within mesolimbic circuits lend further support to the clinical relevance of subcortical system alterations among patients with fibromyalgia as well as patients with chronic back pain. Indeed, as changes in connectivity predict and track with treatment response^43,44^, alterations in corticostriatal and subcortical-striatal circuits in chronic pain may provide crucial information for development and tracking of effective clinical treatments.

As demonstrated in several previous studies in patients with fibromyalgia, relationships exist between altered resting-state fMRI activity and clinical status/affective measures^45–47^. To our knowledge, no prior studies have specifically examined potential relationships between NAcc-MPFC connectivity and various clinical, affective, and cognitive measures in fibromyalgia. As shown in our exploratory correlational analysis within the fibromyalgia group, NAcc-MPFC connectivity was positively correlated with anxiety (STAI-Trait measure). At first glance, the positive correlation is counterintuitive. However, as the overall functional relationship between NAcc and MPFC is reduced in chronic pain, anxiety may be enhancing NAcc-MPFC connectivity in fibromyalgia by engaging MPFC processes of cognition and attention^48,49^. Indeed, the MPFC projects to NAcc and modulates its activity^50,51^—thus, increased NAcc-MPFC connectivity may coincide with enhanced corticostriatal modulation in patients with higher anxiety. In general, striatal dysfunction is related to cognitive deficits and emotional disorders^52–56^. For example, as compared to controls, adolescents with anxiety show hypersensitive striatal response to high-value rewards and greater striatal activity to low-value rewards when unexpected^52^. Complementing such a relationship between dysregulated striatal response and anxiety, our data suggest a relationship between a corticostriatal circuit and anxiety regulation in chronic pain.

As our analyses of NAcc connectivity to other regions within mesolimbic circuits were exploratory, our findings were limited in that they were not corrected for multiple comparisons, and as such should be evaluated prospectively in future investigations. By examining altered connectivity between all 13 regions (see Fig. 3), numerous corticostriatal and subcortical-striatal circuit relationships would be tested. Therefore, future studies would require correction for multiple comparisons and specific testable hypotheses based on our present uncorrected results. Ultimately, in future investigations linking connectivity between brain regions with specific roles in motivation and cognitive processing, relationships between cortico- and subcortical-striatal circuits need to be directly compared in the same patients during resting-state vs. task-based states. Moreover, resting-state alterations may provide valuable mechanistic insight into reward processing sex differences recently observed among chronic pain patients^57^. Future studies examining the extent to which altered corticostriatal resting-state functional connectivity predicts sex-specific altered striatal activity during reward processing may provide critical scaffolding for more carefully tailored chronic pain treatments. In addition, effects of medications need to be considered. Indeed, chronic pain patients on long-term opioid therapy demonstrate disrupted functional connectivity between spinal cord dorsal horns^58^ and within corticostriatal circuits^59^. Notably, the patients included in the present study were opioid naïve (i.e., were not taking opioid medications during the study, nor prior to the study for > 90 days and no lifetime use > 1 month), however, other medications may similarly contribute to individual changes in corticostriatal and/or subcortical-striatal circuits^60^. Finally, through future investigation of how altered striatal circuits influence and interact with other central nervous system (CNS) circuits such as those supporting attention^61^ and expectation^62,63^, the broadening field of functional connectivity may help to clarify the complex neurobiology of chronic pain and thereby potentially aid clinical treatment.

In conclusion, we observed significantly altered corticostriatal and subcortical-striatal circuits in chronic pain during resting-state fMRI. In patients with fibromyalgia vs. healthy controls, significantly reduced NAcc-MPFC connectivity was observed, and within patients, trait anxiety was significantly correlated with corticostriatal connectivity. Together with an exploratory analysis that included resting-state fMRI data from both patients with fibromyalgia and patients with chronic back pain, our results provide evidence of dysregulated corticostriatal circuits connectivity in patients with chronic pain. As underscored by the results from an expanded analysis of subcortical and mesolimbic circuits, widespread brain subcortical-striatal system alterations exist in patients with chronic pain. Ultimately, by providing novel evidence of complex changes within brain corticostriatal and subcortical-striatal circuits across two chronic pain datasets, our results solidify the current foundation of scientific knowledge to support future investigation of clinically meaningful CNS changes in chronic pain.

## Participants and methods

### Participants

Resting-state fMRI data were collected from study participants at Stanford University (from 17 patients with fibromyalgia, 17 healthy individuals (i.e., healthy controls)), and at Duke University (from 17 patients with fibromyalgia and 22 healthy controls). Of these study participants, 1 patient with fibromyalgia was excluded due to a registration issue (i.e., the functional and structural data could not be aligned). In addition, 1 patient with fibromyalgia and 2 healthy controls were excluded due to excessive motion. Thus, a total of 32 patients with fibromyalgia and 37 healthy controls were included in the present study. An additional set of data from a cohort of patients with chronic back pain was acquired from the Open Pain repository (openpain.org). The Open Pain dataset (when accessed in March 2021) contained 34 patients with chronic back pain; data from 3 patients was excluded after preprocessing because their scans could not be aligned (i.e., registered between the functional and structural data).

All patients with fibromyalgia were required to meet the modified American College of Rheumatology 2011 criteria for fibromyalgia: (1) a widespread pain index (WPI) score ≥ 7+ a symptom severity (SS) score ≥ 5, or WPI score 3–6+ SS score ≥ 9; (2) comparable symptoms present for at least 3 months; and (3) no diagnosis that would otherwise explain the pain^64^. Other patient inclusion criteria were: pain in all 4 quadrants of the body, an average pain score of at least 2 (0–10 verbal scale) over the previous month, and no uncontrolled anxiety or depression. Because opioids can have dramatic and long-lasting effects on brain neurophysiology^65,66^, all patients with fibromyalgia were relatively opioid naïve, in that they had not taken any opioid medications within 90 days prior to study participation, and their opioid usage was less than 1 month within their lifetime. Healthy controls reported no history of chronic pain, took no pain medications at the time of the study visit, and did not have depression, anxiety, or major ongoing health conditions.

Ten patients reported taking no mood-altering or pain medications. The 22 remaining patients reported taking nonsteroidal anti-inflammatory drugs (NSAIDs, N = 8), gamma-aminobutyric acid (GABA) analogs (e.g., gabapentin and pregabalin, N = 7), serotonin-norepinephrine reuptake inhibitors (SNRIs, N = 7), tricyclic antidepressants (TCAs, N = 4), selective serotonin reuptake inhibitors (SSRIs, N = 4), low-dose naltrexone (LDN, N = 2), anticonvulsant drugs (N = 2), muscle relaxants (N = 4), other anxiolytics (e.g., buspirone hydrochloride, N = 3), medical cannabis (N = 1), triptans (e.g., rizatriptan, N = 1), other serotonin reuptake inhibitors (e.g., trazodone, N = 1), benzodiazepine (e.g., diazepam, N = 1), and using topical lidocaine patches (N = 1). We retained data from the participant taking medical cannabis in our final analysis as excluding this data did not significantly change the group results.

Thirty-four healthy controls were taking no mood-altering or pain medications. One healthy control participant took gabapentin (100 mg/day) and fluoxetine (40 mg/day) to treat premenstrual symptoms (2 days/month). Another healthy participant took celecoxib (200 mg) 3 weeks prior to study participation due to an ankle injury, and one control took escitalopram (5 mg). We retained data from these 3 participants in our final analysis as excluding them did not significantly affect group results. All participants were required to have no MRI contraindications and not be pregnant or nursing. All study procedures were approved by the Stanford University and Duke University Institutional Review Boards for data collection and analysis, and were performed in accordance with all relevant guidelines and regulations with informed consent obtained from all participants.

Before our analyses, we performed a power analysis for our main study hypothesis to determine the sample size required to detect differences in NAcc-MPFC connectivity between patients with fibromyalgia and healthy controls (OSF, https://osf.io/cj9u8). Figure 3b in Baliki et al.^13^, which described functional connectivity (NAcc-MPFC) between patients with and without persistent pain, served as the input for our power analysis calculations. Results showed that we were well powered to detect group differences such that 17 participants per group would provide ≥ 80% power at alpha = 0.005 to detect a difference in mean connectivity of 0.05 between 2 groups of interest, and we have more than 30 participants in each group.

### General methods

#### Study procedures for patients with fibromyalgia and healthy controls

Data collection was obtained from patients with fibromyalgia and healthy controls following informed consent at the Richard M. Lucas Center for Imaging at Stanford University and at the Duke-UNC Brain Imaging and Analysis Center (BIAC). As part of the study, patients with fibromyalgia and healthy controls completed clinical questionnaires including the Beck Depression Inventory (BDI)^67^, State-Trait Anxiety Inventory (STAI-State, STAI-Trait)^68^, Behavioral Inhibition System/Behavioral Approach System Scales (BIS/BAS)^69^, Profile of Mood States (POMS)^70^, Positive and Negative Affect Schedule (PANAS)^71^, Brief Pain Inventory-Short Form (BPI)^72^, and PROMIS Fatigue (Item Bank v1.0, administered as a computerized adaptive test)^73^. After completing the questionnaires, MRI scans were conducted. Additional questionnaires and brain scans^15,74^ were collected but were not included in the analyses for this study.

### MRI scans for patients with fibromyalgia and healthy controls

#### Dataset from Stanford University

Neuroimaging data were acquired on a 3 T General Electric scanner using an 8-channel head coil (GE Systems, Chicago, IL, USA). One T1 anatomic scan (3D FSPGR [fast spoiled gradient-echo] Irprep BRAVO) was performed for anatomic information. The image covered the whole brain, which included the brainstem and cerebellum, and parameters were as follows: 1-mm slice thickness, 22-mm frequency field of view (FOV), frequency direction anterior/posterior, number of excitations (NEX) 2, flip angle 11°, TR 6.8, TE 2.6, frequency 256, phase 256, and bandwidth 50.00. Functional scans consisted of a Gradient Echo Pulse Sequence using spiral in–out acquisition with 32 oblique slices acquired in a sequential descending slice order. The in-plane resolution was 2.0 mm × 2.0 mm, and slice thickness was 4.0 mm with 0.5-mm slice spacing (TR = 2 s, TE = 30 s, flip angle 76°, pixel size 3.43 mm). By using the spiral in–out scan sequence, orbitofrontal signal drop-out was reduced^75^, and acquisition of medial prefrontal and orbitofrontal cortices was improved. The resting-state scan consisted of 360 volumes.

#### Dataset from Duke University

Scans were conducted on a 3 T GE Premier UHP system with a 48-channel coil at the Duke-UNC Brain Imaging and Analysis Center. The scan sessions consisted of the initial preparatory localizer, asset calibration scans, task fMRI scans^74^, T1 anatomical scan, and a resting-state scan. The fMRI scan parameters were as follows: Gradient Echo Pulse Sequence, echo time (TE) 25 ms, repetition time (TR) 2 s, interleaved slice order, 46 slices, flip angle 77°, 2.9 mm slice thickness, pixel size 2.9 mm. Unlike data collected at Stanford University, a spiral in–out scan sequence was not used in this dataset. A T1 anatomical scan (MPRAGE sequence) was acquired with parameters as follows: whole-brain coverage including the brainstem and cerebellum, 1 mm slice thickness, TR 2.2 s, TE 3.2 ms, 256 mm frequency field of view (FOV), frequency direction anterior/posterior, flip angle 8°. The resting-state scan consisted of 360 volumes.

### Image preprocessing (fibromyalgia and healthy controls)

All resting-state fMRI data were preprocessed using the default preprocessing pipeline in the CONN Toolbox v.20b^76^ running in MATLAB v. R2020a and SPM12. Functional data underwent realignment, and no slice-timing correction was applied, as data from different sites have different slice order information. Outliers were identified based on the observed global blood oxygenation level-dependent (BOLD) fMRI signal and the amount of subject motion. The intermediate outlier identification setting in the CONN toolbox was used to identify framewise displacement (above 0.9 mm or global BOLD signal changes above 5 standard deviations). T1-weighted structural images and functional images were segmented into grey matter (GM), white matter (WM), and cerebrospinal fluid (CSF) tissue classes, and normalized to Montreal Neurologic Institute (MNI) space. Lastly, functional data were smoothed using spatial convolution with a Gaussian kernel of 4 mm full width half maximum.

We then followed CONN’s default denoising pipeline^76^. This step included an anatomic component-based noise correction procedure, corrections for potential confounding effects of WM and CSF, estimated subject-motion parameters, identified outlier scans or scrubbing, and bandpass filtering to 0.008–0.09 Hz. The number of outlier volumes detected ranged from 0 to 18.89% of the total volumes per participant. Rates were comparable to other published analyses^77^.

### Image preprocessing of chronic back pain patients

We analyzed fMRI scan data from the “cbp_resting” project provided by the Open Pain Project (OPP) database (principal investigator: A. Vania Apkarian; http://www.openpain.org; all participants in this dataset consented to participate in the study approved by the Institutional Review Board at Northwestern University.)

The same right NAcc ROI and bilateral MPFC VOI described above were used for NAcc-MPFC connectivity analysis of this comparison dataset. Data were preprocessed using the default preprocessing pipeline in the CONN Toolbox v.20b^76^ running in MATLAB v. R2020a and SPM12. Functional data underwent realignment, but slice-timing correction was not performed. Outliers were identified from the observed global BOLD fMRI signal and the amount of subject motion. The intermediate outlier identification setting in the CONN Toolbox was used to identify framewise displacement. T1-weighted structural images and functional images were segmented into GM, WM, and CSF classes, and normalized to MNI space. Lastly, functional data were smoothed using spatial convolution with a Gaussian kernel of 4 mm full width half maximum. Next, CONN’s default denoising pipeline was followed^76^. The fMRI scan consisted of 244 (14 patients) and 305 volumes (14 patients), and study site(s) information was not reported for the dataset. The number of outlier volumes detected ranged from 0.1 to 14% of the total volumes per participant.

### Corticostriatal connectivity analysis

Based on the approach used in a previous publication^13^, 2 seed regions were defined: the right NAcc and the bilateral MPFC. The right NAcc region of interest (ROI, i.e., subcortical structure defined region) was created from the Desai Atlas included in Analysis of Functional NeuroImages (AFNI)^15^. The size of the bilateral MPFC volume of interest (VOI, i.e., defined by spherical volumes) was fixed at 10-mm diameter spheres centered at (− 4, 50, − 4.5; 4, 50, − 4.5) (Fig. 1A). We used the CONN toolbox for the first-level analysis and group analysis. For first-level analyses, we used the ROI-to-VOI approach. We performed the first-level analysis to extract NAcc-MPFC connectivity per participant. Specifically, we computed a correlation analysis by averaging across the signal in each participant’s NAcc ROI and MPFC VOI, and correlating the signal of their right NAcc with their bilateral MPFC. For the group-level analyses, we compared correlation coefficients for NAcc-MPFC connectivity from each participant to identify differences in connectivity between groups. We also included three covariates in the group-level analyses. As the duration of pain can affect the heterogeneity of functional connectivity^20^ and the datasets were collected at different sites, we included pain duration and study site as covariates. Additionally, due to a significant difference in patients’ age between the two sites, we also included age as covariate (see the “Results” section for details). Next, in exploratory connectivity analyses, we re-tested the primary hypothesis (i.e., connectivity between the right NAcc and bilateral MPFC) using the left NAcc and bilateral NAcc as alternative ROIs to measure connectivity with the bilateral MPFC. For additional exploratory analyses, we used (1) VOIs of different sizes(i.e., 8 mm radius and 10 mm radius bilateral MPFC VOIs centered at [− 4, 50, − 4.5; 4, 50, − 4.5]) and (2) the same MPFC and NAcc VOIs used in a previous study of patients with chronic back pain^13^ (Supplementary Fig. S1).

Within the fibromyalgia patient group, we performed correlational analyses between NAcc-MPFC connectivity and questionnaire measures using the general linear model in the CONN toolbox. Questionnaire measures included pain duration and pain intensity (BPI), affect (PANAS), and fatigue (PROMIS Fatigue). For the correlation analysis, the NAcc-MPFC connectivity data were Fisher-transformed correlation coefficient values from the first-level analysis (i.e., for each subject). We also performed, additional exploratory correlational analyses between NAcc-MPFC connectivity and depression (BDI), anxiety (State-Trait Anxiety), reward-related behavioral inhibition/activation (BIS/BAS Subscales), and total mood disturbance (POMS).

For determining group differences in our primary endpoint of NAcc-MPFC connectivity, we used an unpaired two-sample t-test of correlation coefficients, thresholded for significance at p < 0.05. For the correlational analyses, Bonferroni correction (as described in the “Results” section) was applied to evaluate the significance.

We conducted an additional analysis to compare our NAcc-MPFC connectivity results from patients with fibromyalgia and healthy controls to a separate dataset from a cohort of patients with chronic back pain (from the OPP database, openpain.org). The chronic back pain cohort underwent similar resting-state fMRI, which did not involve any task during the scan. For the analysis across the 3 groups, as in our primary analysis, the same 2 seed regions were used and analysis was conducted using the CONN toolbox (Fig. 1A). Again, we performed a first-level correlation analysis between the right NAcc and bilateral MPFC for each subject’s dataset. Next, using the resulting correlation coefficients from each subject, we compared connectivity between groups using ANCOVA with post-hoc t-tests (Supplementary Fig. S2A). In this analysis, age and pain duration were included as covariates. Study site information was not included as a covariate due to unstated information regarding study site(s) involved in the collection of the chronic back pain dataset.

### Expanded subcortical-striatal connectivity analysis

To further characterize brain subcortical-striatal circuits in chronic pain, we investigated connectivity of the NAcc with other mesolimbic brain regions as additional exploratory VOI-based analyses. The selected regions within mesolimbic circuits included the hippocampus, amygdala, caudate, putamen, anterior cingulate cortex (ACC), insula, thalamus, and ventral pallidum^78^. The coordinates were determined based on previous findings of activation likelihood estimation and parametric voxel-based meta-analysis^23^ (Fig. 2). For this analysis, we again used CONN Toolbox and followed the same statistical analysis steps as described above to compare the right NAcc-subcortical connectivity in fibromyalgia vs. healthy controls (with covariates for pain duration, study site, and age).

In a separate analysis using CONN Toolbox, we evaluated connectivity of the right NAcc with the 12 mesolimbic circuit regions across (1) fibromyalgia vs. chronic back pain, and (2) healthy control vs. chronic back pain (Supplementary Fig. S2B). In addition, we ran a one-way ANCOVA for each of the mesolimbic connections separately (results with uncorrected and FDR corrected p-values are reported in Supplementary Table S3). For the analyses with the chronic back pain dataset, age and pain duration were included as covariates.

## Supplementary Information

Supplementary Information.

## Data Availability

The datasets generated during and/or analyzed during the current study are available from the corresponding author on reasonable request. Raw data for the chronic back pain dataset can be accessed on the Open Pain Project database (http://www.openpain.org).
