# Supplementary material for: Altered resting-state functional connectivity within corticostriatal and subcortical-striatal circuits in chronic pain
Source: Sci Rep. 2022 Jul 25;12:12683. doi: 10.1038/s41598-022-16835-7 (PMC9314446; doi:10.1038/s41598-022-16835-7)
Supplement: Supplementary file 1 — Supplementary Information. [file 41598_2022_16835_MOESM1_ESM.docx]

**Altered resting-state functional connectivity within corticostriatal and subcortical-striatal circuits in chronic pain**

Authors:

Su Hyoun Park, PhD; Anne K. Baker, PhD; Vinit Krishna; Sean C. Mackey, MD. PhD; Katherine T. Martucci, PhD*

**SUPPLEMENTARY FIGURES**


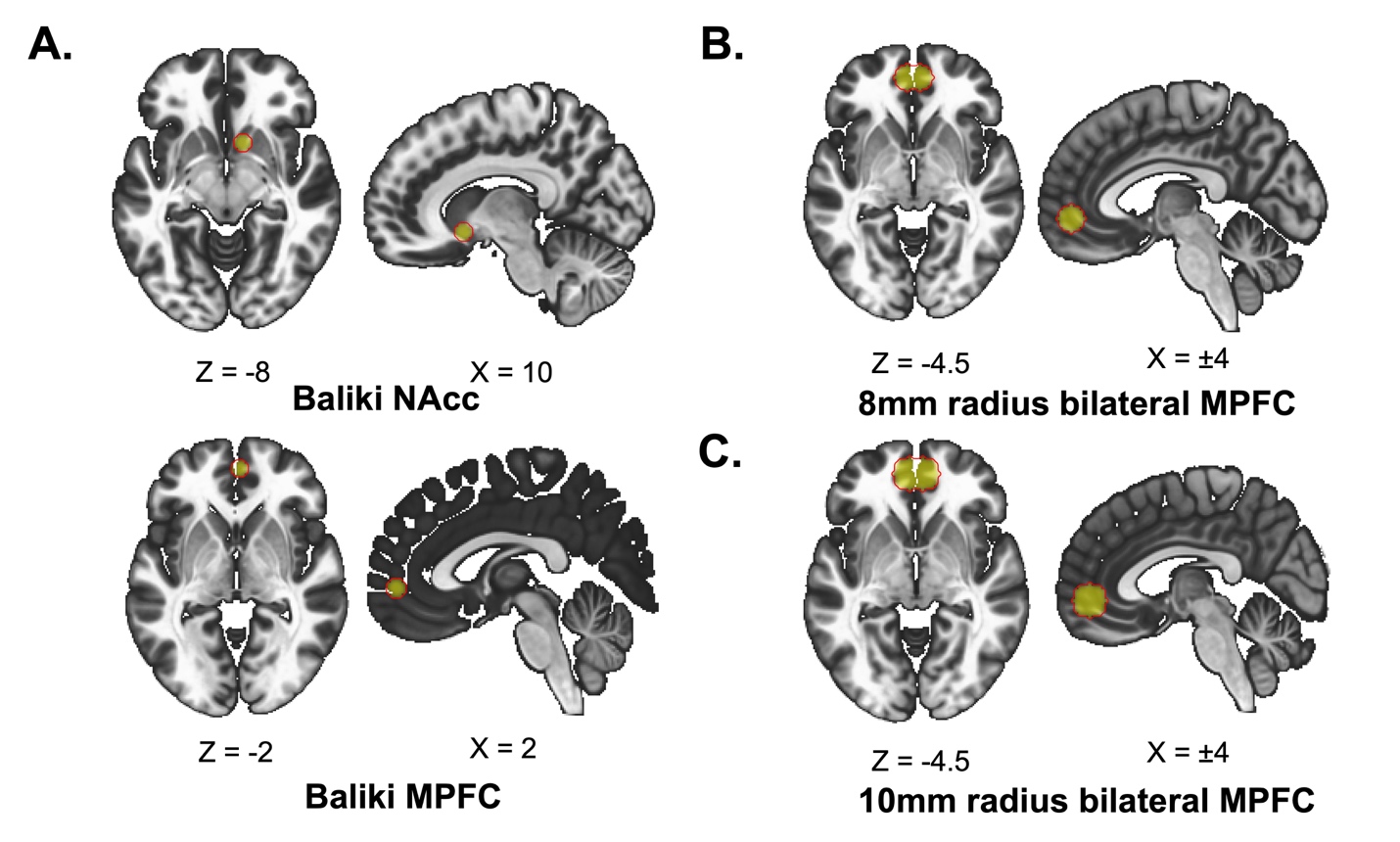


**Supplementary Figure S1. Additional MPFC and NAcc Volumes of Interest (VOIs) for Exploratory Analyses.** A) The VOIs used by Baliki et al. 2012, which include 5-mm radius spheres centered at [10, 12, -8] for the right NAcc VOI (top) and at [2, 52, -2] for the right MPFC VOI (bottom). B) Bilateral MPFC VOI created from partially overlapping spheres centered at [±4, 50, -4.5] each with 8-mm radius. C) Bilateral MPFC VOI created from partially overlapping spheres centered at [±4, 50, -4.5] each with 10-mm radius. Abbreviations: NAcc, nucleus accumbens; MPFC, medial prefrontal cortex.


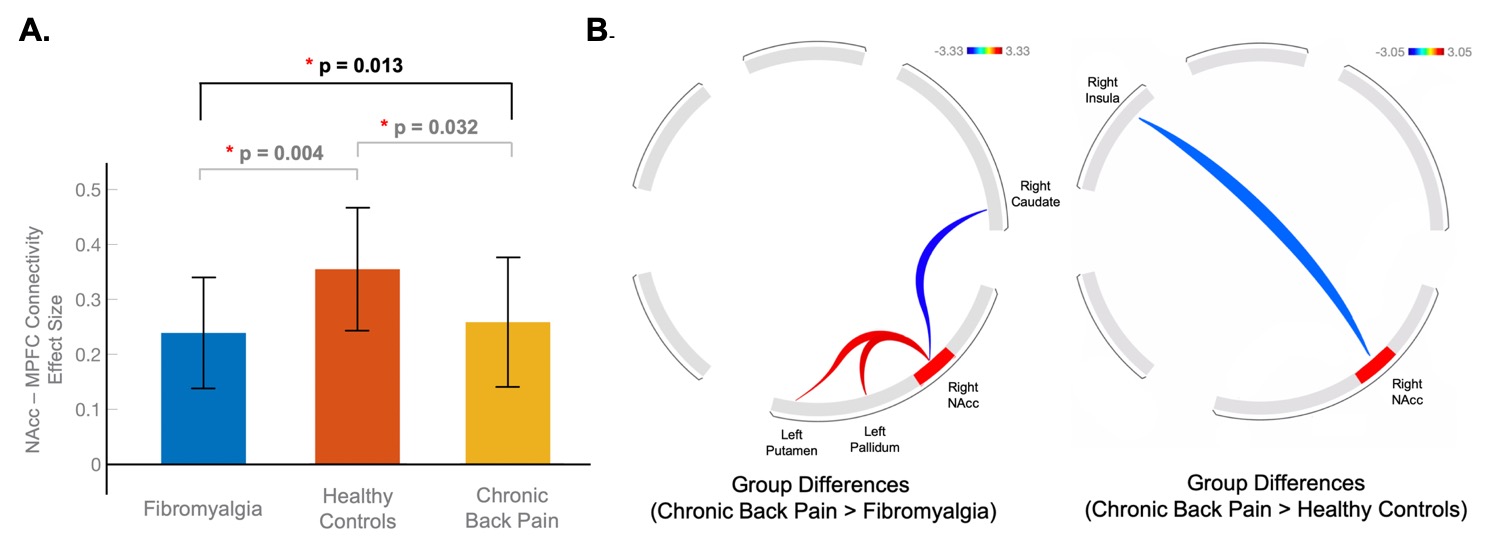


**Supplementary Figure S2.** **Corticostriatal and Mesolimbic Circuit Comparison Across Fibromyalgia vs. Chronic Back Pain vs. Healthy Controls.** A) NAcc-MPFC connectivity (measured as Fischer’s r-to-z transformed correlation coefficients) in patients with fibromyalgia and healthy controls (our dataset) and a separate cohort of patients with chronic back pain from the OPP database (openpain.org). Unlike our primary analysis comparing NAcc-MPFC connectivity between patients with fibromyalgia and healthy controls, only age and pain duration were included as covariates. Study site information was not included as a covariate due to unstated information regarding study site(s) involved in the collection of the chronic back pain dataset. One-way ANCOVA revealed significant effects among the three groups, F(2, 95) = 4.49, p = 0.013. As shown by the post-hoc t-tests, NAcc-MPFC connectivity in patients with chronic back pain and fibromyalgia was significantly decreased as compared to controls (chronic back pain > controls: t(95) = -2.16, p = 0.032; fibromyalgia > controls: t(95) = -2.95, p = 0.004). Error bars indicate the 95% confidence interval. B) Connectivity between the right NAcc and 12 mesolimbic brain regions in chronic back pain vs. fibromyalgia and healthy controls. Left: Compared to fibromyalgia, patients with chronic back pain showed greater connectivity of the right NAcc with the left ventral pallidum (t(59) = 2.75, p-uncorrected = 0.007, p-FDR corrected = 0.047) and left putamen (t(59) = 2.83, p-uncorrected = 0.006, p-FDR corrected = 0.047), but reduced connectivity with the right caudate (t(59) = -2.15, p-uncorrected = 0.03, p-FDR corrected = 0.14). Right: As compared to healthy controls, patients with chronic back pain showed decreased connectivity of the right NAcc with the right insula (t(64) = -2.17, p-uncorrected = 0.033, p-FDR corrected = 0.38). Abbreviations: OPP, Open Pain Project; NAcc, nucleus accumbens; MPFC, medial prefrontal cortex, ANCOVA, analysis of covariance; FDR, false discovery rate.

**SUPPLEMENTARY TABLES**

**Supplementary Table S1. Demographic Information for Patients with Fibromyalgia, Patients with Chronic Back Pain, and Healthy Controls.** Limited demographic information was available on openpain.org for chronic back pain patients. “Other” refers to race other than Asian, Caucasian, African American, Hispanic or Latina, Pacific Islander or Alaskan, or Native American. Education levels are divided into “High School” (up to or through high school), “College/University” (up to or through college/university), and “Advanced Degrees” (post college/university). One patient (fibromyalgia) and 3 healthy controls did not report their income level.

|  | **Stanford University** | | **Duke University** | |  |
| --- | --- | --- | --- | --- | --- |
|  | **Fibromyalgia** | **Healthy Controls** | **Fibromyalgia** | **Healthy Controls** | **Chronic Back Pain** |
| **Total Participants** | **17**  **(all female)** | **17**  **(all female)** | **15**  **(all female)** | **20**  **(all female)** | **31**  **(female N=13)** |
| **Righthanded** | **16** | **16** | **14** | **19** | **31** |
| **Self-Identified Race** | | | | | |
| **American Indian/Alaska Native** | **0** | **0** | **0** | **1** | **N/A** |
| **African American** | **0** | **0** | **2** | **2** |  |
| **Asian** | **2** | **7** | **0** | **0** |  |
| **Caucasian** | **13** | **9** | **12** | **17** |  |
| **Other** | **2** | **1** | **1** | **0** |  |
| **Hispanic or Latina Ethnicity** | **3** | **1** | **1** | **1** |  |
| **Employment Status** | | | | | |
| **Part-time employed** | **3** | **2** | **0** | **1** | **N/A** |
| **Full-time employed** | **6** | **11** | **12** | **14** |  |
| **Unemployed** | **8** | **4** | **3** | **5** |  |
| **Income Level** | | | | | |
| **$0–$29,999** | **5** | **0** | **4** | **3** | **N/A** |
| **$30,000–$59,999** | **3** | **2** | **4** | **5** |  |
| **$60,000 or more** | **8** | **13** | **7** | **11** |  |
| **Education Level** | | | | | |
| **High School** | **3** | **0** | **1** | **0** | **N/A** |
| **College/University** | **11** | **8** | **10** | **13** |  |
| **Advanced Degree** | **3** | **9** | **4** | **7** |  |

**Supplementary Table S2. Age and Questionnaire Measures in Patients with Fibromyalgia and Healthy Controls.** Data from patients with fibromyalgia (N=32) and healthy controls (N=37) are shown for cognitive, affective, and/or behavioral and clinical questionnaires - see text for description of questionnaires used. Due to a few incomplete questionnaires, the number of participants for each measure varies slightly from the total participant counts. Data are presented for descriptive purposes only and therefore the P values shown are not corrected for multiple comparisons. Abbreviations: sd, standard deviation; PANAS, Positive and Negative Affect Schedule; BAS, Behavioral Activation Scale; BIS, Behavioral Inhibition Scale; POMS, Profile of Mood States; PROMIS, Patient-Reported Outcomes Measurement Information System; STAI, State Trait Anxiety Inventory; BDI, Beck Depression Inventory; FAF, Fibromyalgia Assessment Form; BPI, Brief Pain Inventory.

|  | **Fibromyalgia** | | **Healthy Controls** | | **P-Value** |
| --- | --- | --- | --- | --- | --- |
|  | **N** | **Mean±sd** | **N** | **Mean±sd** |  |
| **Age** | 32 | 41.9±12.7 | 37 | 48.4±10.6 | 0.089 |
| **Positive Affect (PANAS)** | 32 | 26±7.3 | 37 | 34.9±6.8 | < 0.001 |
| **Negative Affect (PANAS)** | 32 | 20.4±6.8 | 37 | 14.2±4.4 | < 0.001 |
| **Behavioral Drive (BAS)** | 32 | 11.9±3.8 | 37 | 12.2±3.9 | 0.742 |
| **Behavioral Fun (BAS)** | 32 | 11.5±3.4 | 37 | 12.5±3.4 | 0.231 |
| **Behavioral Reward (BAS)** | 32 | 18.2±4.3 | 37 | 18.0±4.5 | 0.875 |
| **Behavioral Inhibition (BIS)** | 32 | 25.2±6.0 | 37 | 20.4±6.1 | 0.0016 |
| **Total Mood Disturbance (POMS)** | 32 | 21.9±17.7 | 37 | −2.5±8.1 | < 0.001 |
| **Fatigue (PROMIS)** | 32 | 65.5±7.3 | 37 | 46.5±7.9 | < 0.001 |
| **Trait Anxiety (STAI)** | 32 | 49.0±8.3 | 37 | 35.8±9.0 | < 0.001 |
| **State Anxiety (STAI)** | 32 | 40.6±8.4 | 36 | 29.1±7.2 | < 0.001 |
| **Depression (BDI)** | 31 | 16.3±8.8 | 36 | 3.4±5.2 | < 0.001 |
| **Number of Pain Areas (FAF)** | 32 | 13.0±4.0 | 37 | 0.9±1.4 | < 0.001 |
| **Pain Severity (BPI)** | 32 | 5.2±2.0 | 37 | 0.1±0.6 | < 0.001 |
| **Pain Interference (BPI)** | 32 | 22.6±20.9 | 37 | 1.4±4.3 | < 0.001 |

**Supplementary Table S3.** **A One-Way ANCOVA for Each of the Mesolimbic Connections Across Fibromyalgia vs. Chronic Back Pain vs. Healthy Controls.** Results of one-way ANCOVAs between the right NAcc and 12 brain regions within mesolimbic circuits in three groups. Age and pain duration were included as covariates. Abbreviations: ANCOVA, analysis of covariance; NAcc, nucleus accumbens; ACC, anterior cingulate cortex, FDR, false discovery rate.

| **Connectivity between**  **the right NAcc and the:** | **F-Value**  **F(2, 95) =** | **P-uncorrected** | **P-FDR corrected** |
| --- | --- | --- | --- |
| Left Ventral Pallidum | 4.83 | 0.010 | 0.120 |
| Left Putamen | 3.61 | 0.030 | 0.184 |
| Right Caudate | 3.09 | 0.050 | 0.200 |
| Right Amygdala | 2.34 | 0.102 | 0.306 |
| Left Thalamus | 1.47 | 0.235 | 0.433 |
| Right Insula | 1.41 | 0.248 | 0.433 |
| Right ACC | 1.40 | 0.252 | 0.433 |
| ACC | 1.12 | 0.329 | 0.473 |
| Left Insula | 0.96 | 0.385 | 0.473 |
| Left Hippocampus | 0.94 | 0.394 | 0.473 |
| Right Thalamus | 0.80 | 0.452 | 0.493 |
| Left Caudate | 0.01 | 0.993 | 0.993 |
